# Supplementary figures and images for: Glycolytic reprogramming is involved in tissue remodeling on chronic rhinosinusitis
Source: PLoS One. 2023 Feb 16;18(2):e0281640. doi: 10.1371/journal.pone.0281640 (PMC9934430; doi:10.1371/journal.pone.0281640)

Figure 1C

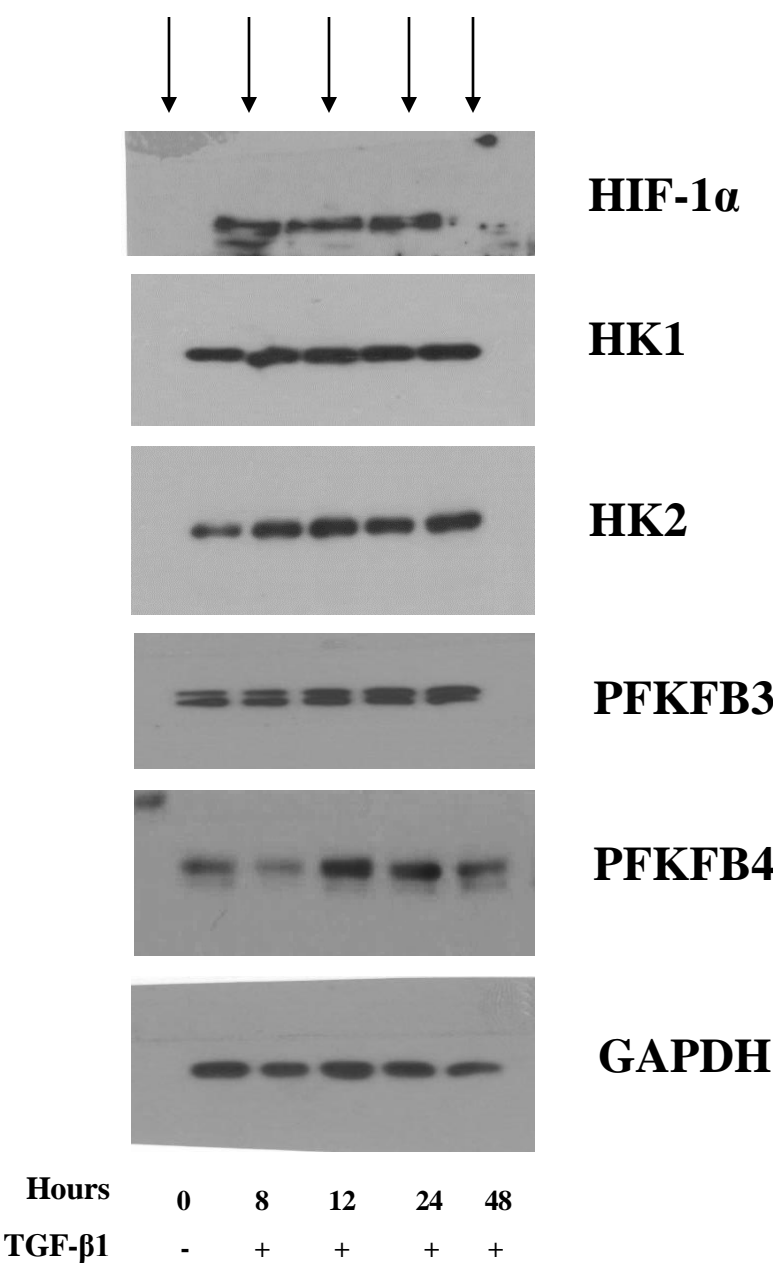

Figure 2B

Figure 2D

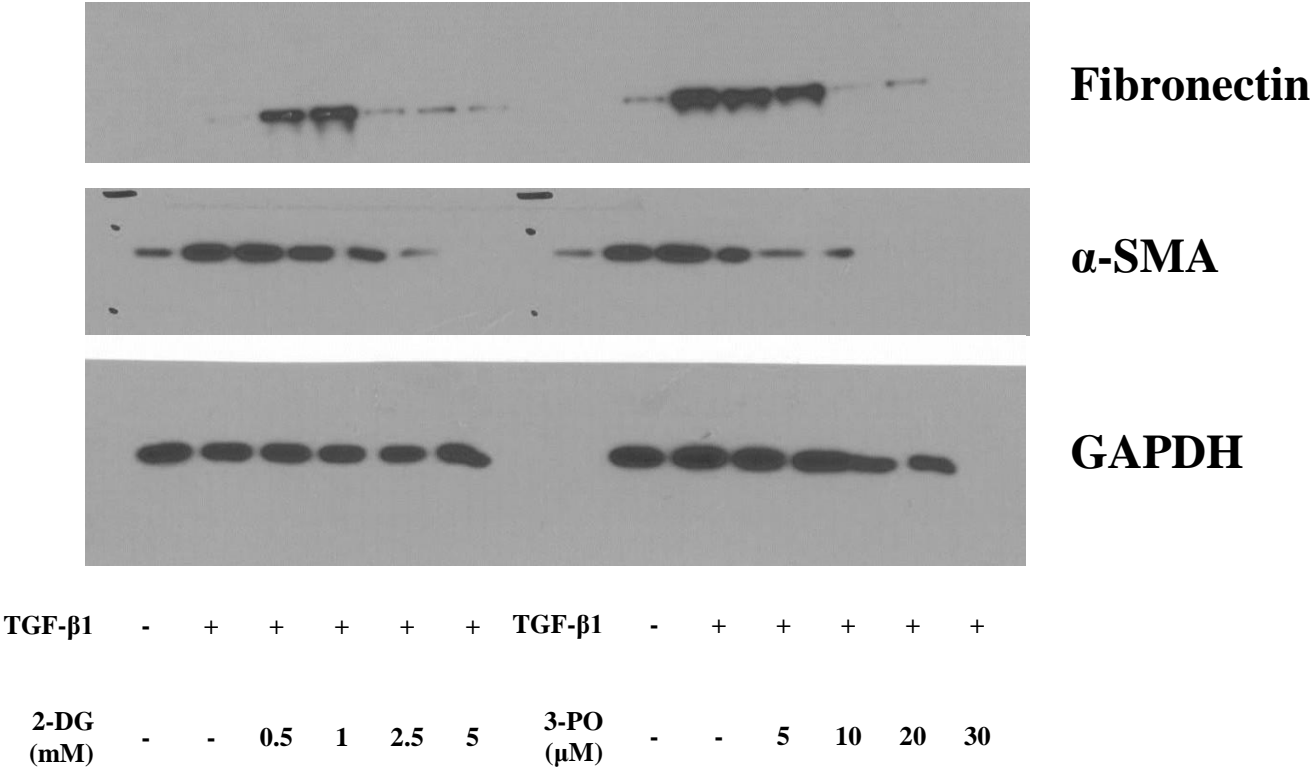

Figure 3B

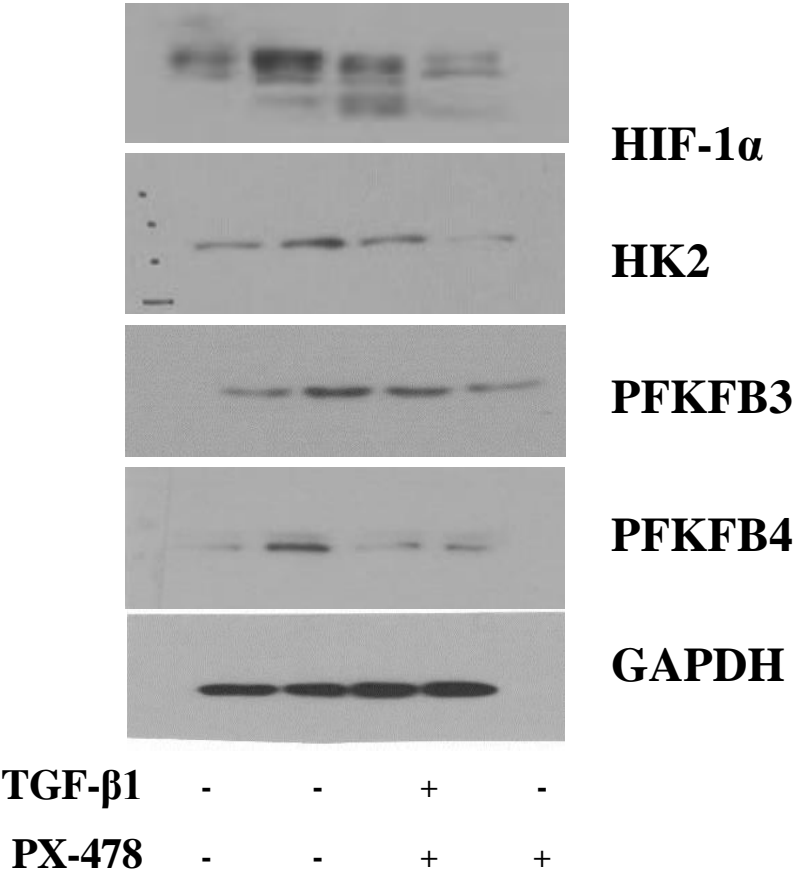

Figure 4B

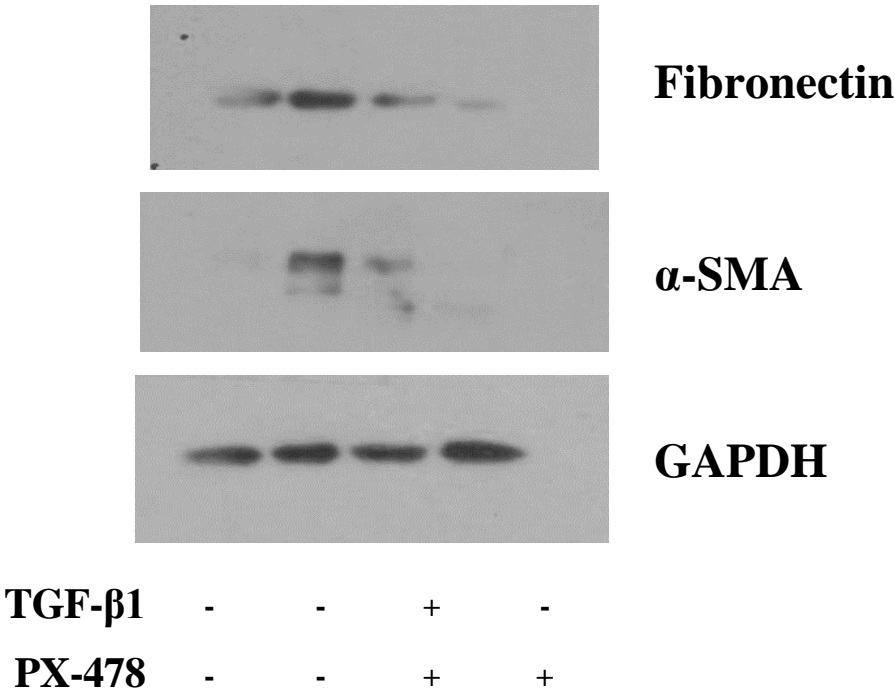

Supplement: S1 Raw images — (PDF) [file pone.0281640.s001.pdf]

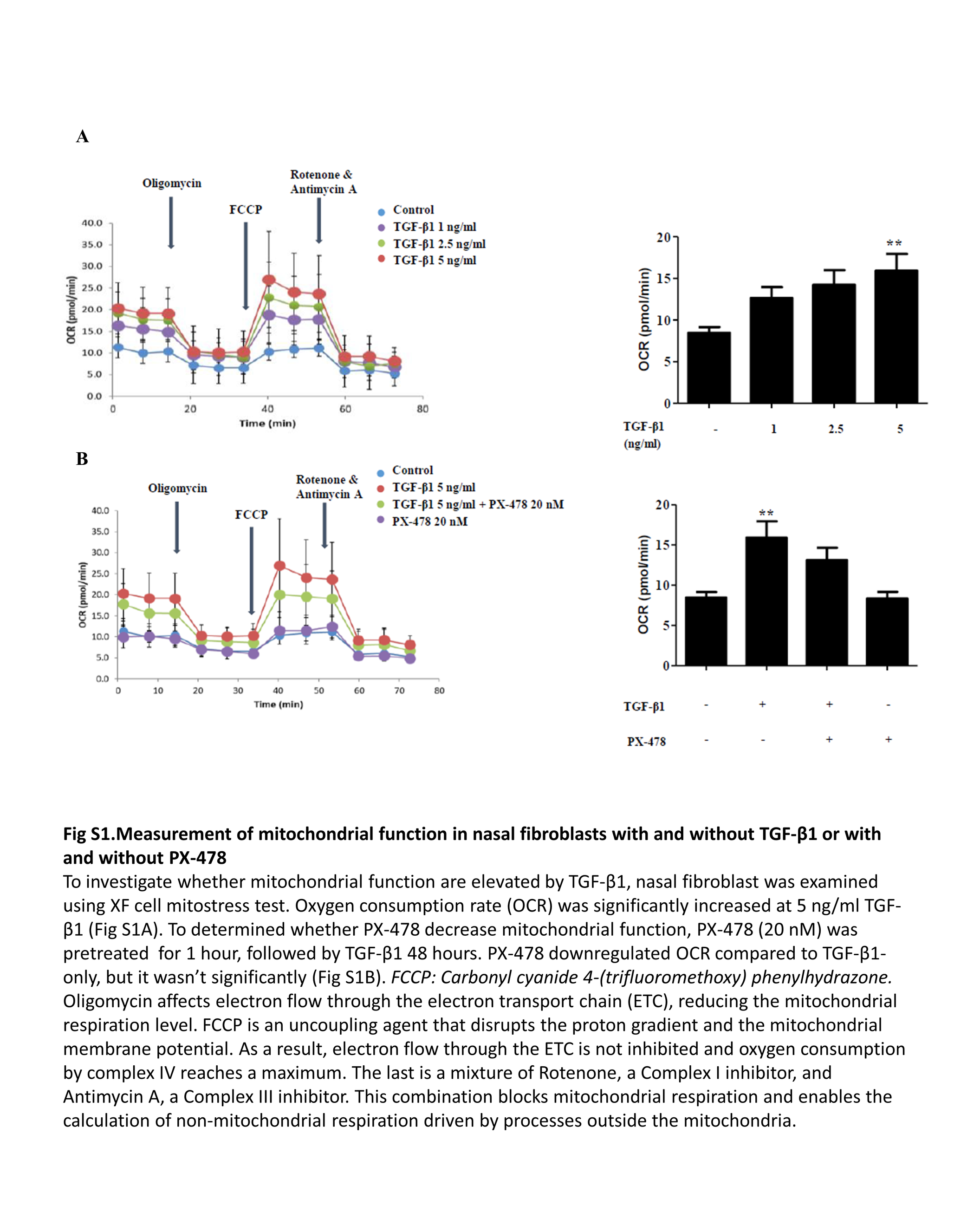

Supplement: S1 Fig — Oxygen consumption rate (OCR) was significantly increased at 5 ng/ml TGF-β1 (S1A Fig). To determined whether PX-478 decrease mitochondrial function, PX-478 (20 nM) was pretreated for 1 hour, followed by TGF-β1 48 hours. PX-478 downregulated OCR compared to TGF-β1- only, but it wasn’t significantly (S1B Fig). FCCP: Carbonyl cyanide 4-(trifluoromethoxy) phenylhydrazone. Oligomycin affects electron flow through the electron transport chain (ETC), reducing the mitochondrial respiration level. FCCP is an uncoupling agent that disrupts the proton gradient and the mitochondrial membrane potential. As a result, electron flow through the ETC is not inhibited and oxygen consumption by complex IV reaches a maximum. The last is a mixture of Rotenone, a Complex I inhibitor, and Antimycin A, a Complex III inhibitor. This combination blocks mitochondrial respiration and enables the calculation of non-mitochondrial respiration driven by processes outside the mitochondria. (TIF) [file pone.0281640.s002.tif]
