# Supplementary material for: Glycolytic reprogramming is involved in tissue remodeling on chronic rhinosinusitis
Source: PLoS One. 2023 Feb 16;18(2):e0281640. doi: 10.1371/journal.pone.0281640 (PMC9934430; doi:10.1371/journal.pone.0281640)
Supplement: Attachment — Submitted filename: Response to Reviewers.docx [file pone.0281640.s003.docx]

Title: Glycolytic reprogramming is involved in tissue remodeling on chronic rhinosinusitis

Corresponding author: Il-Ho Park, Jae-Min Shin

**Dear Reviewers**

Thank you for sending us many good comments about the study. We sincerely responded to the opinions of you. I've attached a response to the comments below.

**Review #1**

**Major comments:**

**1. All Western blotting bands need to be quantitatively analyzed. In addition, the bands of GAPDH shown in Figure 1C are connected, indicating that the loading amount of the protein was too high.**

- Thank you for your comments. We quantified all western blot data and added them to the figures. GAPDH data have been corrected.

**2. What does ECAR refer to in all the figures, basic glycolysis, glycolysis capacity, or glycolysis reserve**?

- Glycolysis is the process by which glucose is converted to pyruvate and ATP is produced. The XF Glycolysis Stress Test presents the measure of glycolysis as the ECAR rate reached by a given cell after the addition of saturating amounts of glucose. The first injection is a saturating concentration of glucose. The cells utilize the glucose injection and catabolize it through the glycolytic pathway to pyruvate, producing ATP, NADH, water, and protons. The extrusion of protons into the surrounding medium causes a rapid increase in ECAR. This glucose-induced response is reported as the rate of glycolysis under basal conditions. The second injection is oligomycin, an ATP synthase inhibitor. Oligomycin inhibits mitochondrial ATP production, and shifts the energy production to glycolysis, with the subsequent increase in ECAR revealing the cellular maximum glycolytic capacity. The final injection is 2-deoxy-glucose (2-DG), a glucose analog that inhibits glycolysis through competitive binding to glucose hexokinase, the first enzyme in the glycolytic pathway. The resulting decrease in ECAR confirms that the ECAR produced in the experiment is due to glycolysis. The difference between glycolytic capacity and glycolysis rate defines glycolytic reserve. ECAR, prior to glucose injection, is referred to as nonglycolytic acidification; caused by processes in the cell other than glycolysis.

• Glycolytic capacity: This measurement is the maximum ECAR rate reached by a cell following the addition of oligomycin, effectively shutting down oxidative phosphorylation and driving the cell to use glycolysis to its maximum capacity.

• Glycolytic reserve: This measure indicates the capability of a cell to respond to an energetic demand as well as how close the glycolytic function is to the cell’s theoretical maximum.

• Nonglycolytic acidification (basal levels): This measures other sources of extracellular acidification that are not attributed to glycolysis

We have added these details to materials & methods for the reader's understanding.

**3. In Figure 3D, TGFβ group and TGFβ+PX-478 group had no significant increase of ECAR after giving glucose. Please explain it.**

- In Figure 3D, TGF-β1 significantly upregulated the level of ECAR, and in the TGFβ+PX-478 group, glucose-induced ECAR upregulation was suppressed. This means that PX-478 inhibits cell reactivity by Glucose, that is, glycolysis. We demonstrated in Fig. 3 that PX-478 inhibits glycolysis-mediated factors HIF-1a, HK2, PFKFB3 and PFKFB4. Therefore, the level of ECAR did not increase significantly despite the administration of the substrate glucose.

* Glucose administered at 15 min time-dependently increased the level of ECAR. (Red Graph)

**4. The authors stated that they studied the role of glycolytic reprogram in CRS. However, they only focused on glycolysis, and provided no data regarding oxidative phosphorylation.**

- Thanks for your comment. We used the XF substrate oxidation stress test kit to confirm the oxidative phosphorylation reaction and attach the relevant data as a supplement. This test method can measure the oxidative phosphorylation reaction to substrates such as glucose and pyruvic acid by evaluating the change in oxygen consumption of living cells. As a result, TGF-β1 increased the overall level of OCR, and PX-478 partially suppressed it.

**5. The authors should compare the difference between CRSsNP and CRSwNP.**

- CRS is divided into CRS without nasal polyp (CRSsNP) and CRS with nasal polyp (CRSwNP) according to the presence or absence of nasal polyp. It was reported that extracellular matrix deposition, fibrosis, massive immune cell infiltration were observed in the stroma of CRSsNP, and fibrin deposition, edema were observed in CRSwNP. The original intention of the study was to classify CRS into only CRSsNP and CRSwNP to see the difference in glycolytic reprogramming of fibroblasts. In fact, CRS is classified into Th1, Th2, and Th 17 types according to the immune response, and subclassified into eosinophilic and non-eosinophilic according to the infiltrated immune cells, so it was difficult to find a tendency in classification based on nasal polyp. Therefore, there was a limit to the detailed classification of the recruited patients. So the study was conducted based on CRS and normal subjects. In the future, we plan to subdivide the patient group according to the immune response and conduct research.

**6. Chen et al recently studied glycolysis in CRS epithelial cells and its involvement in the pathogenesis of CRS (https://doi.org/10.1016/j.jaci.2022.09.036). This recent study should be compared and discussed.**

- Thanks for your comment. We reviewed the papers you mentioned and additionally described their relevance to our research in the discussion section. Thank you.

**Minor comment:**

**1. On line 354, the format of the reference was different from the others.**

- We revised the reference style.

**Reviewer #2**

**1. Did the authors study any cytokines both proinflammatory and anti-inflammatory.**

- Thank you for your nice comments. As a follow-up study, we are conducting a study on the correlation between glycolytic reprogramming and proinflammatory cytokines, IL-6 and IL-8. In previous studies, we demonstrated that environmental hazardous substances such as Asian sand dust and diesel exhaust particles stimulate nasal fibroblasts to secrete proinflammatory cytokines such as IL-6 and IL-8 [1, 2]. Therefore, in the future, we plan to conduct a study on the role of glycolysis in the process of producing IL-6 and IL-8 by these external antigens.

**2. In ECAR experiment did the authors had done normalization of data. If not please include in revised manuscript**

- Thank you for your comment. ECAR experiments are conducted in two steps. First, equilibration is performed through a calibrant utility plate, and then the sensor cartridge is loaded to measure drug treatment and result values. Experimental data was normalized using Wave 2.6 software provided by Agilent. Normalized data were included in Figure 1A and 3D. All values shown in the graph are normalized values. Additionally, cell count was performed before measuring ECAR values, and through this, ECAR values were normalized. Relevant information will be attached to the manuscript.

**3. The figures are not clear enough to read. Please include high quality figures with font size that is clearly visible and readable.**

- Thank you for your comment. We replaced all figures as high quality figures.

**References**

1.	Yang, H.-W., J.-H. Park, J.-M. Shin, H.-M. Lee, and I.-H. Park, *Asian Sand Dust Upregulates IL-6 and IL-8 via ROS, JNK, ERK, and CREB Signaling in Human Nasal Fibroblasts.* American Journal of Rhinology & Allergy, 2019. **34**(2): p. 249-261.

2.	Kim, J.A., J.H. Cho, I.-H. Park, J.-M. Shin, S.-A. Lee, and H.-M. Lee, *Diesel Exhaust Particles Upregulate Interleukins IL-6 and IL-8 in Nasal Fibroblasts.* PLOS ONE, 2016. **11**(6): p. e0157058.
